# Transcriptome Analysis Reveals the Regulation of *Aureobasidium pullulans* under Different pH Stress

**DOI:** 10.3390/ijms242216103

**Published:** 2023-11-09

**Authors:** Kai Zhang, Wan Wang, Qian Yang

**Affiliations:** 1 School of Life Science and Technology, Harbin Institute of Technology, Harbin 150006, China; 2 State Key Laboratory of Urban Water Resources and Environment, Harbin Institute of Technology, Harbin 150090, China

**Keywords:** *Aureobasidium pullulans*, pH, transcriptome

## Abstract

*Aureobasidium pullulans* (*A. pullulans*), a commonly found yeast-like fungus, exhibits adaptability to a wide range of pH environments. However, the specific mechanisms and regulatory pathways through which *A. pullulans* respond to external pH remain to be fully understood. In this study, we first sequenced the whole genome of *A. pullulans* using Nanopore technology and generated a circle map. Subsequently, we explored the biomass, pullulan production, melanin production, and polymalic acid production of *A. pullulans* when cultivated at different pH levels. We selected pH 4.0, pH 7.0, and pH 10.0 to represent acidic, neutral, and alkaline environments, respectively, and examined the morphological characteristics of *A. pullulans* using SEM and TEM. Our observations revealed that *A. pullulans* predominantly exhibited hyphal growth with thicker cell walls under acidic conditions. In neutral environments, it primarily displayed thick-walled spores and yeast-like cells, while in alkaline conditions, it mainly assumed an elongated yeast-like cell morphology. Additionally, transcriptome analysis unveiled that *A. pullulans* orchestrates its response to shifts in environmental pH by modulating its cellular morphology and the expression of genes involved in pullulan, melanin, and polymalic acid synthesis. This research enhances the understanding of how *A. pullulans* regulates itself in diverse pH settings and offers valuable guidance for developing and applying engineered strains.

## 1. Introduction

*Aureobasidium pullulans* (*A. pullulans*), also known as “black yeast” due to its melanin synthesis during growth, is a yeast-like fungus belonging to the ascomycete group [1,2]. The life cycle of *A. pullulans* is intricate, and its cell morphology varies under different nutrient media or culture conditions, including yeast-like cells, chlamydospores, and hyphal forms [3,4,5]. Strains of *A. pullulans* thrive in diverse environments such as soil, bee nests, seawater, freshwater, plant surfaces, wood, and even extreme conditions like polar regions, polluted waters, and arid deserts, showcasing its remarkable adaptability to complex environmental conditions [6,7,8,9]. Because of its broad distribution, robust adaptability, high tolerance, short life cycle, strong reproductive capacity, and the production of numerous extracellular or intracellular metabolic products during fermentation, *A. pullulans* has increasingly piqued the interest of scientists and researchers [10,11]. With the advancement and widespread use of high-throughput sequencing and gene editing technologies, scientists have embarked on research into the biosynthesis and regulation of valuable metabolic products within *A. pullulans*, opening up possibilities for various applications.

*A. pullulans*, found in diverse ecological settings, has developed its unique adaptation mechanisms through long-term evolution. During the fermentation and cultivation, it produces a wide array of highly active and valuable fermentation products, including pullulan, polymalic acid (PMA), gluconic acid, melanin, liamocin, fructooligosaccharides, lipase, and more [12,13,14,15,16]. These metabolic products are currently employed or show tremendous potential across various domains within the biotechnology industry. Previous research has uncovered key genes and enzymes involved in the synthesis of these compounds. For example, glucokinase (Gluk), phosphoglucomutase (Pgm), UDP-glucose pyrophosphorylase (Ugp), multidomain α-glucan synthase 2 (Amags2), pullulan synthase (Pul1), Glycogenin (Glg), and Glucosyltransferase (Fks) have been identified as contributors to pullulan biosynthesis [1,10]. Meanwhile, Pks, Cmr1, Scd1, and Thr1 play essential roles in melanin synthesis [14]. Additionally, Pyc, Mdh, and Mls1 serve as critical enzymes in the PMA synthesis process [12].

The ability of microorganisms to adapt to changes in their environment is crucial for their survival, and one key factor that affects microbial growth and metabolism is environmental pH [17,18]. Currently, research on microbial homeostatic mechanisms of pH can be categorized into three main areas. The first category involves the transport or uptake of H^+^ ions to maintain intracellular pH stability. For example, alkaliphilic bacteria can maintain stable intracellular pH by exchanging monovalent cations like Na^+^ and Li^+^ with external H^+^ ions through antiport proteins [19]. The second category focuses on microorganisms’ ability to adjust the environmental pH by modulating metabolic products. They can change the acidity or alkalinity of their surroundings by producing organic acids or alkaline substances. These metabolic products can neutralize acidic or alkaline components in the environment, thus maintaining pH balance inside and outside the cells [20]. Additionally, microorganisms can adapt to changing environmental pH by regulating the expression of specific genes. Various signaling pathways, including the calcineurin pathway [21], PKA pathway [22], Pacc-Pal signal pathway [23], and other stress signaling pathways, have evolved to regulate external pH. These pathways can initiate or inhibit the transcription and translation of specific genes, leading to the production of proteins or other molecules that assist microorganisms in adapting to and coping with fluctuations in environmental pH. *A. pullulans*, known for its ability to thrive in a wide range of pH conditions, undoubtedly possesses unique metabolic pathways and regulatory mechanisms. Previous research has shown that pH plays a role in the morphological changes in *A. pullulans* cells. Under low pH conditions, most cells take on a filamentous morphology, resulting in lower pullulan production but rapid biomass growth. Conversely, under mildly neutral pH conditions, most cells exhibit a yeast-like morphology, which is the primary cell type involved in polysaccharide synthesis [1,5]. However, the specific mechanisms and regulatory pathways through which *A. pullulans* responds to external pH and its adaptation strategies remain to be fully understood.

To gain deeper insights into how *A. pullulans* responds to changes in external pH environments, this study first obtained the complete genomic information of *A. pullulans* through genome sequencing. Next, the study examined the effects of varying environmental pH on the growth, development, cell morphology, and secondary metabolism of *A. pullulans*. Finally, transcriptome analysis was conducted to investigate patterns of gene expression in *A. pullulans* under acidic, neutral, and alkaline conditions, followed by a differential gene expression analysis. This comprehensive approach aims to enhance our understanding of the processes involved in the growth, development, and regulation of secondary metabolism in *A. pullulans* under different pH conditions. This research not only contributes to establishing a solid theoretical foundation for the study of *A. pullulans* but also provides valuable guidance for the design and application of engineered strains.

## 2. Results

### 2.1. Genome Circular Map

**Figure 1:**
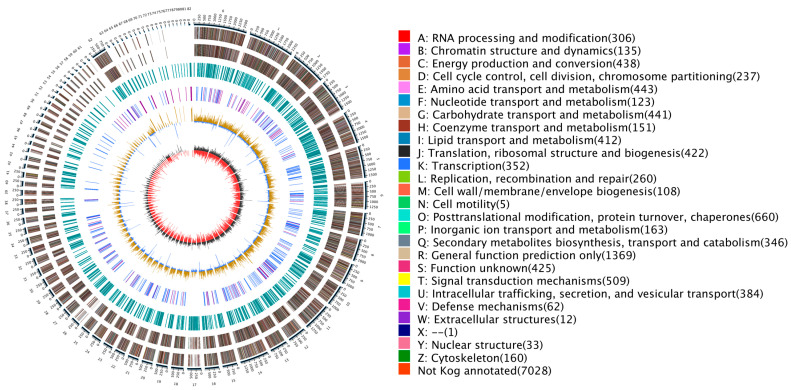
Represents a genome circular map of *A. pullulans.* The outermost circle indicates the genome’s size. The second and third circles represent genes on the positive and negative strands of the genome, respectively, with different colors denoting various functional classifications of KOG. The fourth circle illustrates repetitive sequences. The fifth circle displays tRNA and rRNA, with tRNA in blue and rRNA in purple. The sixth circle denotes the GC content, with light yellow indicating regions with GC content higher than the genome’s average GC content. Higher peaks represent greater differences from the average GC content, while blue areas represent regions with GC content lower than the genome’s average. The innermost circle displays GC-skew, where dark gray represents regions with a higher G content than C, and red represents regions with a higher C content than G.

Using the third-generation sequencing technology of Nanopore, DNA samples of *A. pullulans* were sequenced, and the obtained clean reads were assembled using NECAT 1.12 software [12]. Consequently, the genome size of *A. pullulans* was determined to be 38,314,936 bp, with a GC content of 49.99%. The total length of coding genes was 27,339,078, with a total CDS (coding sequence) length of 20,557,863 and a total of 37,383 CDS. Based on this genomic information, a circular diagram (Figure 1) was generated. The outermost circle indicates the genome’s size. The second and third circles represent genes on the positive and negative strands of the genome, respectively, with different colors denoting various functional classifications of KOG. The fourth circle illustrates repetitive sequences. The fifth circle displays tRNA and rRNA, with tRNA in blue and rRNA in purple. The sixth circle denotes the GC content, with light yellow indicating regions with GC content higher than the genome’s average GC content. Higher peaks represent greater differences from the average GC content, while blue areas represent regions with GC content lower than the genome’s average. The innermost circle displays GC-skew, where dark gray represents regions with a higher G content than C, and red represents regions with a higher C content than G.

### 2.2. The Influence of Different Days on Products of A. pullulans

**Figure 2:**
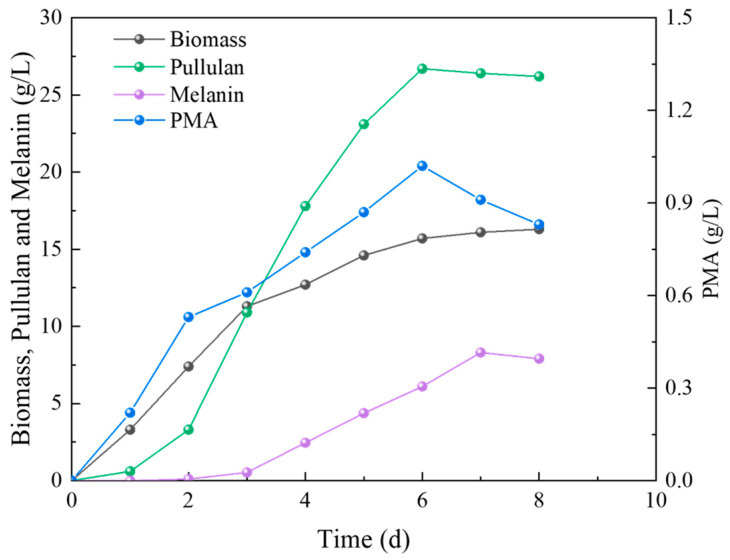
Impact of varied fermentation days on the biomass and product.

During the fermentation experiments conducted in 5 L airlift fermenters, 200 mL samples were collected every 24 h to measure the biomass of *A. pullulans* and the production of secondary metabolites. The results are depicted in Figure 2. It can be observed that the biomass consistently increased over time, with a relatively rapid increase in the initial three days. Pullulan production exhibited an increasing trend starting from the second day and reached its peak of 26.7 g/L on the sixth day, followed by a minor decline as the fermentation continued. Melanin synthesis occurred later than pullulan synthesis and had a lower yield. It displayed an upward trend within the 3–7 day cultivation period, with the maximum melanin synthesis observed at 8.3 g/L on the seventh day. Although PMA was relatively low, its presence was detected on the first day, and its production exhibited an upward trend in the first six days, reaching a peak of 1.02 g/L.

### 2.3. The Influence of Different pH Levels on the Product

**Figure 3:**
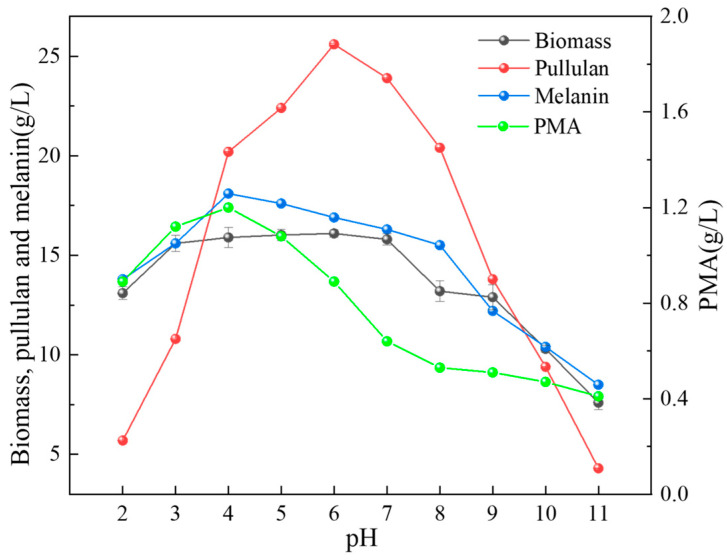
Effect of initial pH on biomass and metabolite production in *A. pullulans*.

To assess the impact of the initial pH of the culture medium on the biomass and metabolite production of *A. pullulans*, fermentation media with pH values ranging from 2 to 11 were chosen for a 6 day cultivation period. It was observed that both excessively acidic and alkaline conditions had the most restrained influence on pullulan synthesis (Figure 3). Under pH 2 and pH 11, the pullulan yields were only 5.7 g/L and 4.3 g/L, respectively, which were notably lower compared to the optimal pH of 6, where it reached 25.6 g/L. Regarding biomass, it was evident that *A. pullulans* could thrive in a broad pH range, with substantial proliferation occurring within the pH range of 3–7. However, alkaline conditions inhibited its growth. PMA and melanin exhibited similar trends, both reaching their peak synthesis levels at pH 3, with values of 1.2 g/L and 18.1 g/L, respectively.

### 2.4. Observing the Morphology of A. pullulans at Different pH Levels

**Figure 4:**
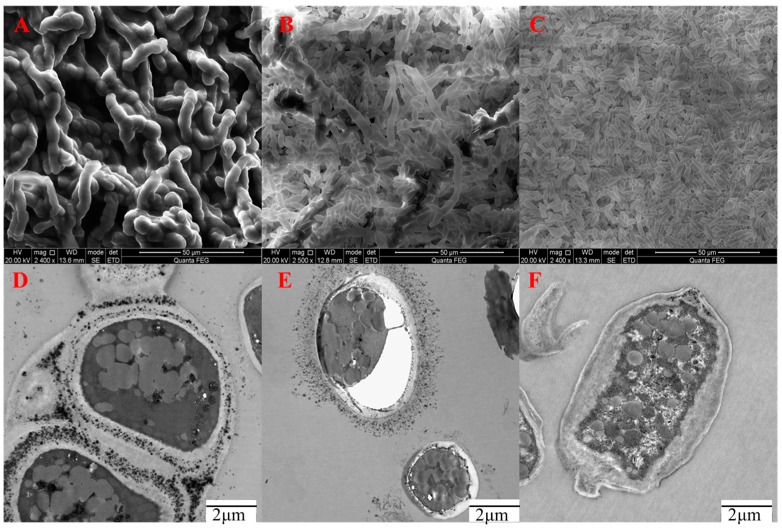
SEM and TEM images of *A. pullulans* at different pH levels. (**A**): SEM of pH 4.0; (**B**): SEM of pH 7.0; (**C**): SEM of pH 10.0; (**D**): TEM of pH 4.0; (**E**): TEM of pH 7.0; (**F**): TEM of pH 10.0.

To understand how pH influences the microscopic morphology of *A. pullulans*, we selected pH levels of 4.0, 7.0, and 10.0 to represent acidic, neutral, and alkaline environments, respectively, based on differences in biomass and metabolite production. We used SEM and TEM to examine the microscopic morphology of *A. pullulans*. Under pH 4.0, SEM images revealed that most of the cells appeared filamentous and had a thick-walled chlamydospore-like appearance with a darker color. The cells were enveloped by polysaccharides, giving them a viscous appearance (Figure 4A). Additionally, TEM images showed a higher number of polysaccharides and melanin attached to the cell wall of the filamentous cells, resulting in a darker intracellular color (Figure 4D). In the pH 7.0 environment, SEM observations showed the presence of filamentous cells, thick-walled chlamydospores, and yeast-like cells, with only a small fraction of cells exhibiting a darker color (Figure 4B). The cell walls were thinner, and there were lower amounts of polysaccharides and melanin compared to the acidic environment. Large vacuoles were also observed within the cells (Figure 4E). In the alkaline environment of pH 10.0, *A. pullulans* primarily existed as elongated yeast-like cells (Figure 4C). While the cell walls were thicker, there was less melanin adhering to them, with melanin being distributed more within the cells. Additionally, the cells in the alkaline environment were longer than those in the acidic and neutral environments.

### 2.5. Gene Expression Analysis

**Figure 5:**
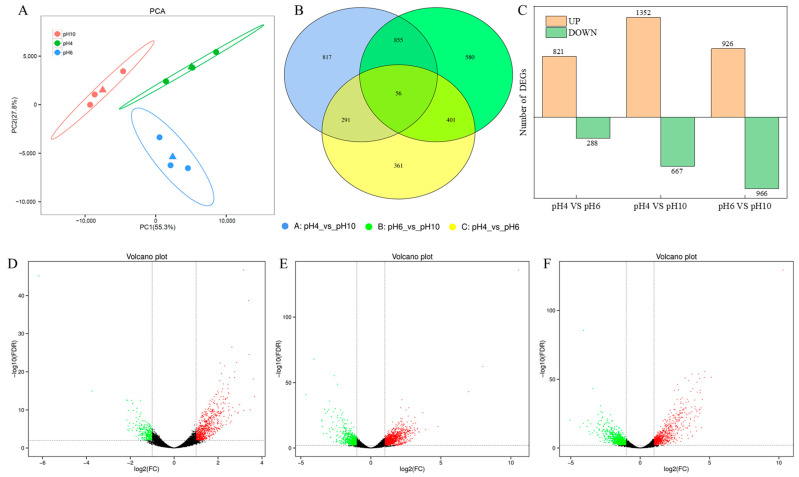
Gene expression analysis. (**A**): PCA analysis of gene expression between samples; (**B**): Venn diagram analysis; (**C**): number of differentially expressed genes; (**D**): volcano plot of pH 4.0 vs. pH 7.0; (**E**): volcano plot of pH 4.0 vs. pH 10.0; (**F**): volcano plot of pH 7.0 vs. pH 10.0. In (**D**–**F**), the green dots represent down-regulated differentially expressed genes, the red dots represent up-regulated differentially expressed genes, and the black dots represent non-differentially expressed genes).

In Figure 5A, a PCA analysis of gene expression between samples is presented. The three samples from the pH 4.0, pH 7.0, and pH 10.0 groups are closely clustered, indicating a high degree of similarity between the samples. Figure 5B illustrates a Venn diagram showing the overlap of differentially expressed genes between different comparison groups. Specifically, there are 1109 differentially expressed genes in the pH 4.0 vs. pH 7.0 comparison, 2019 in the pH 4.0 vs. pH 10.0 comparison, and 1892 in the pH 7.0 vs. pH 10.0 comparison, with a total of 56 differentially expressed genes common to all three comparison groups. Figure 5C provides a statistical summary of the number of differentially expressed genes in each of the three comparison groups. Figure 5D–F depicts volcano plots for the comparison groups of pH 4.0 vs. pH 7.0, pH 4.0 vs. pH 10.0, and pH 7.0 vs. pH 10.0, respectively. In the pH 4.0 vs. pH 7.0 comparison (Figure 5D), 821 genes are upregulated, while 288 genes are downregulated. In the pH 4.0 vs. pH 10.0 comparison (Figure 5E), 1352 genes are upregulated and 667 genes are downregulated. Finally, in the pH 7.0 vs. pH 10.0 comparison (Figure 5F), 926 genes are upregulated and 966 genes are downregulated.

### 2.6. Differential Gene Expression Analysis

**Figure 6:**
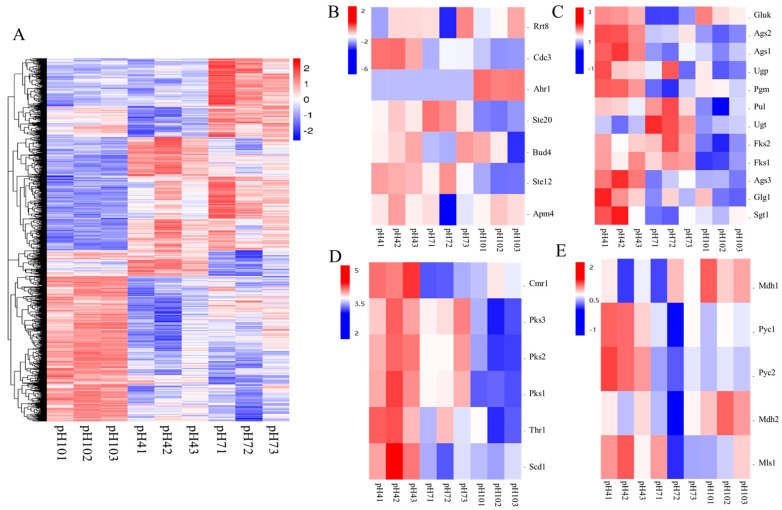
Clustering analysis of differential gene expression. (**A**) heat map of all differential gene expression levels; (**B**) heat map of gene expression levels related to growth, development and differentiation; (**C**) heat map of gene expression levels related to pullulan synthesis; (**D**); heat map of gene expression levels related to melanin synthesis; (**E**) heat map of gene expression levels related to PMA synthesis.

This study has generated a heatmap illustrating the differential gene expression (Figure 6A) based on the variance in gene expression levels in *A. pullulans* under different pH conditions. Furthermore, to gain a deeper understanding of how *A. pullulans* regulates its growth, development, and the synthesis of secondary metabolites under varying pH conditions, we have created heatmaps depicting the expression levels of genes related to growth and development (Figure 6B), as well as those involved in pullulan synthesis (Figure 6C), melanin synthesis (Figure 6D), and PMA synthesis (Figure 6E). Ste12, Ste20, Ahr1, and Apm4 are responsible for regulating mycelial growth and development, while Bud4 and Rrt8, respectively, control bud-site selection and spore cell wall assembly. In Figure 6B, we observed that, except for the upregulation of the Ahr1 gene under alkaline conditions, the expression levels of other genes related to hyphal growth and spore formation were downregulated. Concerning pullulans synthesis, except for Gluk, which exhibited reduced expression under neutral conditions, the expression of other genes was higher under acidic conditions. Genes associated with melanin synthesis exhibited higher expression under acidic conditions compared to alkaline conditions. Regarding PMA synthesis-related genes, Mdh1 and Mdh2 showed higher expression under alkaline conditions, while Pyc1, Pyc2, and Msl1 displayed higher expression under acidic conditions.

### 2.7. Functional Annotation of Differentially Expressed Genes

The CDS sequences obtained from transcriptome sequencing were subjected to comparative analysis using the NR, Swiss-Prot, Pfam, COG, GO, and KEGG databases. The functional information of differentially expressed genes was then compiled and summarized based on the annotations retrieved from these databases. In the comparison groups of pH 4.0 vs. pH 7.0, pH 4.0 vs. pH 10.0, and pH 7.0 vs. pH 10.0, a total of 1019, 1952, and 1756 genes, respectively, were successfully annotated. Specific details regarding the annotations in each comparison group can be found in Table 1.

#### 2.7.1. GO Analysis

**Figure 7:**
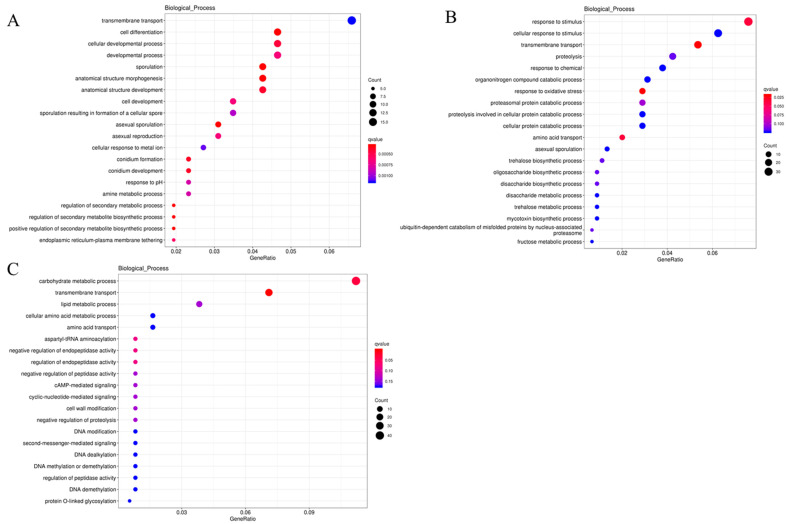
Enrichment of biological processes in differentially expressed genes. (**A**) enrichment of biological processes in pH 4.0 vs. pH 7.0; (**B**) enrichment of biological processes in pH 4.0 vs. pH 10.0; (**C**) enrichment of biological processes in pH 7.0 vs. pH 10.0.

A pathway analysis of differentially expressed genes allows us to gain insights into the significantly altered metabolic pathways under experimental conditions. Through the enrichment analysis of GO functions associated with differentially expressed genes, we observed the following patterns: In the comparison group of pH 4.0 vs. pH 7.0 (Figure 7A), biological processes were mainly centered around transmembrane transport, cell differentiation, cellular developmental processes, developmental processes, sporulation, and, to a lesser extent, regulation of secondary metabolic processes, regulation of secondary metabolite biosynthetic processes, and positive regulation of secondary metabolite biosynthetic processes. Additionally, there was evidence of a response to pH. In the comparison group of pH 4.0 vs. pH 10.0 (Figure 7B), biological processes were primarily related to responses to stimuli, cellular responses to stimuli, transmembrane transport, proteolysis, and responses to chemicals, with some involvement in trehalose biosynthetic and metabolic processes, oligosaccharide biosynthetic and metabolic processes, general metabolic processes, and fructose metabolic processes. In the comparison group of pH 7.0 vs. pH 10.0 (Figure 7C), biological processes were mainly associated with carbohydrate metabolic processes, transmembrane transport, lipid metabolic processes, cellular amino acid metabolic processes, amino acid transport, and, to a lesser extent, DNA modification, regulation of peptidase activity, and regulation of peptidase activity.

#### 2.7.2. KEGG Analysis

**Figure 8:**
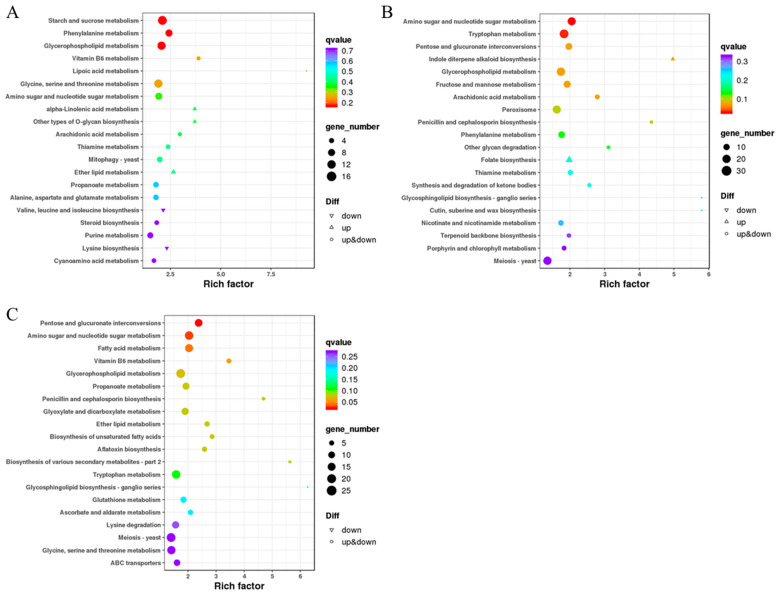
KEGG analysis of differentially expressed genes. (**A**) KEGG analysis in pH 4.0 vs. pH 7.0; (**B**) KEGG analysis in pH 4.0 vs. pH 10.0; (**C**) KEGG analysis in pH 7.0 vs. pH 10.0.

The growth, development, and synthesis of fungal products are orchestrated by a complex network of metabolic pathways. Conducting pathway enrichment analysis on differentially expressed genes can provide insights into the functions of these metabolic pathways and their interconnections. In the comparison group of pH 4.0 vs. pH 7.0, the top 20 enriched pathways, as depicted in Figure 8A, include starch and sucrose metabolism, glycerophospholipid metabolism, and glycine, serine, and threonine metabolism, each involving more than 15 differentially expressed genes. Furthermore, pathways such as phenylalanine metabolism, as well as amino sugar and nucleotide sugar metabolism, comprise over ten differentially expressed genes. In the comparison group of pH 4.0 vs. pH 10.0, illustrated in Figure 8B, the top 20 enriched pathways contain more than 20 genes in pathways like amino sugar and nucleotide sugar metabolism, tryptophan metabolism, glycerophospholipid metabolism, peroxisome, and Meiosis—yeast. Notably, indole diterpene alkaloid biosynthesis and folate biosynthesis are enriched pathways with upregulated genes, while the glycosphingolipid biosynthesis ganglio series is an enriched downregulated pathway. In the comparison group of pH 7.0 vs. pH 10.0, shown in Figure 8C, the enriched pathways comprise over 20 differentially expressed genes in pathways such as Pentose and glucuronate interconversions, amino sugar and nucleotide sugar metabolism, glycerophospholipid metabolism, Tryptophan metabolism, Meiosis—yeast, and glycine, serine, and threonine metabolism. In addition, the glycosphingolipid biosynthesis ganglio series is enriched in downregulated pathways only.

## 3. Discussion

Fungal adaptation to environmental pH is not only dependent on maintaining intracellular pH stability and osmotic pressure but is also associated with the synthesis of secondary metabolites [24,25,26]. Therefore, understanding the adaptive mechanisms of *A. pullulans* to environmental pH is of significant research importance. It not only aids in dissecting signaling pathways and downstream targets from a fundamental research perspective but also guides the design of strains with varying pH tolerance to enable the production of target metabolites under harsh fermentation conditions. In this study, we obtained the complete genome sequence of *A. pullulans* using Nanopore Technologies and created a circular diagram. This provides a foundation for subsequent research at the gene level of *A. pullulans*. Subsequently, we investigated the impact of fermentation time on biomass and the synthesis of secondary metabolites. From Figure 2, we can observe that early growth primarily involves cell proliferation, with the synthesis of secondary metabolites gradually starting from the second day. Among these metabolites, pullulans are the major product, reaching a maximum of 26.7 g/L, which is more than three times the amount of melanin produced. Most microorganisms maintain a narrow pH range for cytoplasmic, typically favoring a neutral to slightly acidic pH environment [27]. To adapt to different pH conditions, fungal cells regulate environmental pH during growth by secreting small molecules, ensuring ion homeostasis inside and outside the cells to support hyphal growth [28]. Although *A. pullulans* can survive in environments with a pH range of 2 to 11, there are significant differences in biomass and the synthesis of secondary metabolites due to variations in environmental pH. We can see that neutral conditions are optimal for growth (Figure 3). Typically, when fungi encounter environments with lower pH than their optimum, they adjust by secreting ammonia-like substances, raising the external pH to the favorable range. Acidophilic fungi, on the other hand, often secrete organic acids to lower the environmental pH when growing in alkaline conditions [29,30]. For example, *Candida albicans* can break down amino acids as carbon sources and convert them into ammonia, which is released outside the cell, thereby increasing the external pH [31]. Under alkaline pH conditions, pathways related to glucose metabolism produce more acidic substances [32]. Moreover, high pH environments reduce pigment production and induce oxidative stress [33], which is one of the reasons for the decrease in melanin production under alkaline conditions (Figure 3); this is consistent with the downregulation of gene expression related to melanin synthesis in Figure 6D. In addition, slightly acidic conditions are more favorable for PMA production, while a slightly neutral environment is more suitable for pullulan production. This is because the cell membrane and cell wall can maintain the relative stability of the intracellular environment, ensuring normal cell proliferation [34]. This prompts *A. pullulans* to differentiate into hyphae and use polysaccharides to form thicker cell walls for protection (Figure 4A,D). Acidic conditions also lead to the hydrolysis of pullulans, resulting in lower pullulan production compared to neutral conditions. Under alkaline conditions, all three metabolite productions are lower due to the inhibition of Aureobasidium pullulan growth, subsequently affecting the synthesis of secondary metabolites.

**Figure 9:**
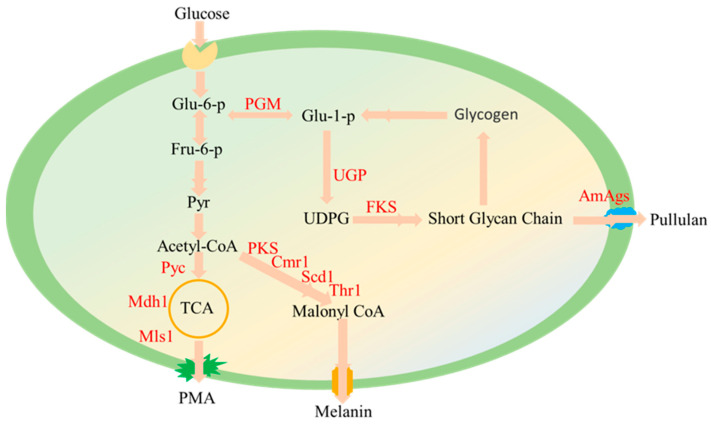
Schematic diagram of pullulan, melanin, and PMA biosynthesis pathways.

The environmental pH significantly influences fungal cell growth, differentiation, and secondary metabolism [35]. To elucidate the genetic basis of the observed variations in morphology and metabolite production in different pH environments, this study conducted a transcriptome analysis under pH conditions of 4.0, 7.0, and 10.0, representing acidity, neutrality, and alkalinity, respectively. This study discovered that *A. pullulans* exhibits similarities to Yarrowia lipolytica [36,37], notably the elongated yeast-like cellular morphology in alkaline conditions (Figure 4). This adaptation might be linked to the upregulation of the Ahr1 gene under alkaline environmental conditions. Furthermore, when comparing biological processes between pH 4.0 and pH 7.0, we observed that transmembrane transport, cell differentiation, sporulation, and secondary metabolite synthesis were the predominant biological processes under acidic conditions (Figure 7A). Particularly in the pH 7.0 vs. pH 10.0 comparison, over 70 genes were enriched in carbohydrate metabolic processes and transmembrane transport (Figure 7C). This explains why pullulans and melanin are produced more abundantly under neutral to slightly acidic conditions. When comparing acidic conditions to other pH levels, this study identified biological processes related to pH adaptation and responses to external stimuli. Notably, in the pH 4 vs. pH 10 comparison, multiple processes related to oligosaccharide biosynthesis and trehalose biosynthesis were enriched (Figure 7B). Oligosaccharides and trehalose are known to mitigate the adverse effects of extreme environmental conditions [38]. Additionally, fungi typically regulate pH by secreting ammonia-like compounds under acidic conditions. The secretion of these compounds is primarily driven by the breakdown of amino acids. This mechanism, observed in typical fungi such as *colletotrichum* and *fusarium oxysporum*, contributes to the elevation of environmental pH [29,39]. We also observed this phenomenon in *A. pullulans*. For instance, pathways including Glycine, serine, and threonine metabolism, alanine, aspartate, and glutamate metabolism, as well as valine, leucine, and isoleucine biosynthesis, were enriched under acidic conditions (Figure 8A). This reflects one of the adaptive strategies employed by the fungus to cope with acidic environments. Finally, this study has provided schematic representations of the pullulan, melanin, and PMA synthesis pathways (Figure 9). These pathways initiate from glu-6-p and subsequently diverge, with one branch dedicated to pullulan synthesis and the other leading to the production of acetyl coenzyme A. The latter is then utilized for melanin and PMA synthesis.

## 4. Materials and Methods

### 4.1. Strains and Materials

Strain *A. pullulans* HIT-LCY^3^ was isolated and preserved in the laboratory [3]. YPD (glucose 20.0 g/L, yeast extract 10.0 g/L, tryptone 20.0 g/L). All other reagents were analytically pure.

### 4.2. Genome Analysis

The genome sequencing experiment was conducted following the protocol provided by Oxford Nanopore Technologies (ONT) [40]. To extract high-quality genomic DNA, purity, concentration, and integrity were assessed using Nanodrop, Qubit, and 0.35% agarose gel electrophoresis. Subsequently, the BluePippin automated nucleic acid recovery system was employed to recover large DNA fragments. Library construction was performed using the SQK-LSK109 ligation kit, involving DNA damage repair, end repair, and magnetic bead purification. Subsequently, adapter ligation and another round of magnetic bead purification were carried out. The resulting library was quantified using Qubit. Finally, the library was subjected to sequencing.

### 4.3. The Impact of Fermentation Time on Products of A. pullulans

Strains preserved in a −80 °C freezer were inoculated into a seed culture medium. After one day of cultivation at 28 °C, they were transferred to a 5 L airlift fermenter with a 5% inoculation rate, and the aeration rate was maintained at 0.2 vvm. Every 24 h, 200 mL of the fermentation broth was sampled to assess biomass, pullulan production, melanin production, and PMA production [1,11,13]. For biomass extraction, cells were centrifuged at 5000 rpm for 10 min. The resulting cells were dried at 80 °C until a constant weight was achieved. To extract pullulan, twice the volume of ethanol was added to 100 mL of supernatant and stood for 12 h. The mixture was then centrifuged at 12,000 rpm for 10 min. The resulting precipitate was dried at 80 °C until a constant weight was achieved. Melanin was extracted by mixing 50 mL of supernatant with 1 mol/L hydrochloric acid to adjust the pH to 3. After vigorous shaking, the mixture was placed at 60 °C to induce complete precipitation. The product was filtered through a 600-mesh filter paper and dried at 60 °C until a constant weight was achieved. For PMA extraction, 2 mL of the obtained supernatant was added to 1 mL of 2M H_2_SO_4_ and incubated at 85 °C for 8 h. After neutralization, the hydrolyzed sample was analyzed using high-performance liquid chromatography with a C18-EP organic acid column. The analysis was performed at 40 °C using 5 mM H_2_SO_4_ at a flow rate of 0.6 mL/min to determine the content of malic acid and calculate the yield of PMA.

### 4.4. Influence of Different pH Levels on Product Formation

Prepared solutions of 1 mol/L NaOH and HCl were sterilized, and the pH of the fermentation medium was subsequently adjusted to 3, 4, 5, 6, 7, 8, 9, 10, and 11, respectively. Following these adjustments, the cultures were incubated for six days to assess biomass, pullulan production, melanin production, and PMA production.

### 4.5. Observing the Morphology of A. pullulans at Different pH Levels

pH levels of 4.0, 7.0, and 10.0 were chosen to represent acidic, neutral, and alkaline environments, respectively. Initially, cells were cultured in a YPD medium at pH 7.0 and 28 °C, with agitation at 180 rpm for 24 h. Subsequently, cells equivalent to 10% of the culture volume were transferred to a YPD medium adjusted to pH 4.0, 7.0, and 10.0, respectively. After six days, cell phenotypes at different pH values were observed under an optical microscope and photographed. Following centrifugation at 12,000 rpm, the collected cells were fixed with 50% glutaraldehyde for 30 min and then dehydrated in a series of ethanol concentrations: 50%, 75%, 90%, and 100%, each for 10 min. Subsequently, acetone was used for dehydration, followed by freeze-drying at −70 °C. The dried samples were evenly spread on conductive adhesive and subjected to gold coating before being observed under an SEM [4]. For the centrifuged cells, they were washed in 0.1 M phosphate buffer at pH 7.4 (containing 0.1–0.5 mol/L cysteine) for 15–30 min. Then, they were fixed with 6% glutaraldehyde and stored at 4 °C for 2 h. Afterward, the fixative solution was discarded, and the samples were rinsed three times with 0.1 M phosphate buffer at pH 7.0 for 15 min each time. Next, the samples were fixed with 1% osmic acid solution for 1–2 h. Following this, the excess osmic acid solution was removed, and the samples were rinsed three times with 0.1 M phosphate buffer at pH 7.0 for 15 min each time. Subsequently, the samples underwent a dehydration process with a gradient of ethanol concentrations (including 30%, 50%, 70%, 80%, 90%, and 95%) with each concentration treatment lasting for 15 min, followed by a final treatment with 100% ethanol for 20 min. Finally, the samples were transitioned to pure acetone for 20 min. The samples were infiltrated with a mixture of embedding agent and acetone (V/V = 1/1) for 1 h, followed by a mixture of embedding agent and acetone (V/V = 3/1) for 3 h, and then pure embedding agent overnight. Post embedding, the samples were heated at 70 °C overnight to achieve proper embedding. These embedded samples were sliced into sections of 70–90 nm thickness using an ultramicrotome, followed by staining with lead citrate and uranyl acetate in 50% ethanol solution. The sections were subsequently observed using a TEM [41].

### 4.6. Transcriptome Analysis

Eukaryotic mRNA was enriched using magnetic beads containing Oligo (dT), and state-of-the-art molecular biology equipment was employed to assess the purity, concentration, and integrity of RNA samples. Once library qualification was confirmed, sequencing was conducted on the Illumina platform. Genomic annotation was performed utilizing proprietary databases such as NR, KOG, COG, KEGG, Swissprot, and GO, among others. Subsequent analyses included differential gene expression analysis, differential gene function annotation, and functional enrichment analysis, which were all based on gene expression levels across different comparison groups [42].

### 4.7. Statistical Analysis

All data were statistically analyzed using SPSS 19.0 (IBM Corporation, Armonk, NY, USA). Duncan’s multiple-range test was used to compare data means. *p* < 0.05 was considered statistically significant.

## 5. Conclusions

In this study, we first determined that the optimal fermentation time for *A. pullulans* was six days. Subsequently, we explored the impact of different pH values on the biomass and synthesis of secondary metabolites in *A. pullulans*. Through SEM and TEM observations, this study observed that under acidic conditions, the fungus primarily exhibited a filamentous morphology with thicker cell walls. Under neutral conditions, thick-walled chlamydospores were predominant, but hyphae and yeast-like cells were also present. Conversely, under alkaline conditions, elongated yeast-like cells were the predominant morphology. Furthermore, transcriptome analysis revealed that *A. pullulans* can adjust its response to external pH changes by regulating metabolic pathways of amino acid, oligosaccharide and trehalose, sugar, and pathways related to responses to stimulation. This study establishes a solid theoretical foundation for the research of *A. pullulans* and offers valuable insights for the development and application of engineered strains in biotechnology.

## Figures and Tables

**Table 1:** Statistic of annotation results.

Database/Number	pH 4.0 vs. pH 7.0	pH 4.0 vs. pH 10.0	pH 7.0 vs. pH 10.0
GO	652	1331	1132
KEGG	479	1021	862
KOG	354	695	602
Pfam	680	1408	1183
Swissprot	452	886	731
NR	1019	1923	1756
Total	1019	1952	1756

## Data Availability

Where no new data were created.
